# Back-to-monomer recycling of polycondensation polymers: opportunities for chemicals and enzymes

**DOI:** 10.1039/d1ra08217e

**Published:** 2022-01-05

**Authors:** Shanmugam Thiyagarajan, Evelien Maaskant-Reilink, Tom A. Ewing, Mattijs K. Julsing, Jacco van Haveren

**Affiliations:** Wageningen Food & Biobased Research Wageningen P. O. Box 17 6700 AA The Netherlands shanmugam.thiyagarajan@wur.nl

## Abstract

The use of plastics in a wide range of applications has grown substantially over recent decades, resulting in enormous growth in production volumes to meet demand. Though a wide range of biomass-derived chemicals and materials are available on the market, the production volumes of such renewable alternatives are currently not sufficient to replace their fossil-based analogues due to various factors, in particular cost-effectiveness. Hence, the majority of plastics are still industrially produced from fossil-based feedstocks. Moreover, various reports have clearly raised concern about the plastics that are not recycled at their end-of-life and instead end up in landfills or the oceans. To avoid further pollution of our planet, it is highly desirable to develop recycling processes that use plastic waste as feedstock. Chemical recycling processes could potentially offer a solution, since they afford monomers from which new polymers can be produced, with the same performance as virgin plastics. In this manuscript, the opportunities for using either chemical or biochemical (*i.e.*, enzymatic) approaches in the depolymerization of polycondensation polymers for recycling purposes are reviewed. Our aim is to highlight the strategies that have been developed so far to break down plastic waste into monomers, providing the first step in the development of chemical recycling processes for plastic waste, and to create a renewed awareness of the need to valorize plastic waste by efficiently transforming it into virgin plastics.

## Abstract 2

This review highlights the promising strategies developed to break down polycondensate polymers back to monomers using chemicals and enzymes. Our aim is to create a renewed awareness to valorize plastic waste into virgin plastics.

## Graphical abstract

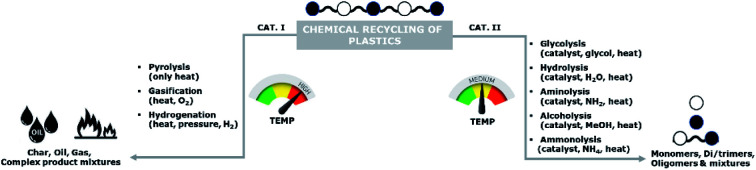

## Introduction

To lower greenhouse gas emissions and simultaneously reduce the accumulation of plastics in nature, a transition towards a sustainable circular economy is foreseen. This transition necessitates an increased use of renewable carbon feedstocks, derived from biomass or CO_2_, for the production of chemical building blocks and materials. Furthermore, in order to close the loop, it will require reuse of materials to the greatest possible extent. To achieve this, polymeric materials must be reused or recycled at the end of their life. Especially for synthetic, non-biodegradable polymers, recycling is a must to mitigate CO_2_ emissions associated with their production & waste management and accumulation of plastic waste in nature. In addition, reuse of plastic material supports the objective to become less dependent on finite fossil resources. The global production of plastics increased from 2 million tonnes in 1950 to more than 350 million tonnes today, with a market growth rate of >5% per annum. Projections are that plastic production may increase up to more than 1200 million tonnes in 2050. From 1950 to 2015, an estimated amount of 4.900 million tonnes of synthetic polymers was discarded or landfilled, while at ∼600 million tonnes, only a minor part of the used plastics was recycled.^1^ Additionally, Ryberg *et al.* estimated that 6.2 million tonnes of macroplastics are leaked into nature annually.^2^ The massive amount of carbon present in synthetic polymers and their accumulation in nature demonstrate the urgency of the plastic waste problem and at the same time the huge potential used polymers have as a feedstock for new materials and other products in a circular economy.

Recycling of synthetic polymers can be achieved by reuse of a product (*i.e.* PET-bottles) or mechanical recycling to a new product. However, this cannot be continued indefinitely as the quality of the product will drop with each step either due to degradation or the accumulation of contaminants. In addition, mechanically recycled plastics are often not approved in certain applications, such as food packaging.^3^ This currently imposes limits to the percentage of synthetic polymers that can be recycled. In order to increase the reuse of resources and circumvent the loss of value, back to monomer recycling enables the use of plastic fractions that are not or no longer suitable for mechanical recycling. Depolymerization of synthetic polymers to their constituent monomeric building blocks allow repolymerisation into a high-value product.

Synthetic polymers essentially can be classified as either polymers in which monomers are linked *via* C–C bonds such as polyolefins, polystyrene, synthetic rubber and polyacrylates or as polycondensation polymers, which contain hydrolysable bonds between the constituent monomers. This review gives an overview of back-to-monomer degradation (breakdown) technologies for polycondensation polymers with a significant market volume and discusses their potential for industrial application. Chemical as well as enzymatic methods will be discussed. Both methods will be reviewed and compared for different polycondensation polymers. We believe the transition to a circular economy will entail moving away from the use of polyolefins towards polycondensates due to their greater potential for recycling opportunities using back-to-monomer/oligomers strategies and (in some cases) biodegradation.

## Chemical recycling

Currently, the recycling of plastics can be conducted using various methods: primary (mechanical or closed-loop), secondary (mechanical or open-loop), tertiary (chemical or feedstock), and quaternary (energy recovery or waste valorisation).^4,5^ Among these methods, primary and secondary recycling are the most commonly applied, however, chemical recycling has also received significant attention in the past decade.

**Scheme 1:**
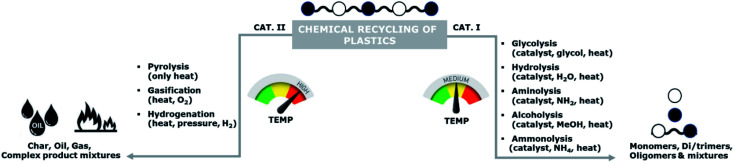
Classification of chemical recycling of plastics.

Chemical recycling can be divided into two main categories: (i) processes involving mild temperatures and solvents of which the resulting products are mainly oligomers and monomers, and (ii) high temperatures processes of which the products are a mixture of char, oils, and gases (Scheme 1). Examples of these high temperature processes are pyrolysis, gasification, and hydrogenation.

The high temperature processes typically result in complex mixtures of crude oils, which might be used for chemical synthesis.^7–10^ However, re-use of recovered monomers for polymerization requires very high purity levels, which are typically not feasible to obtain. Hence, these high temperature processes are unsuitable as methods for back-to-monomer recycling.

**Fig. 1:**
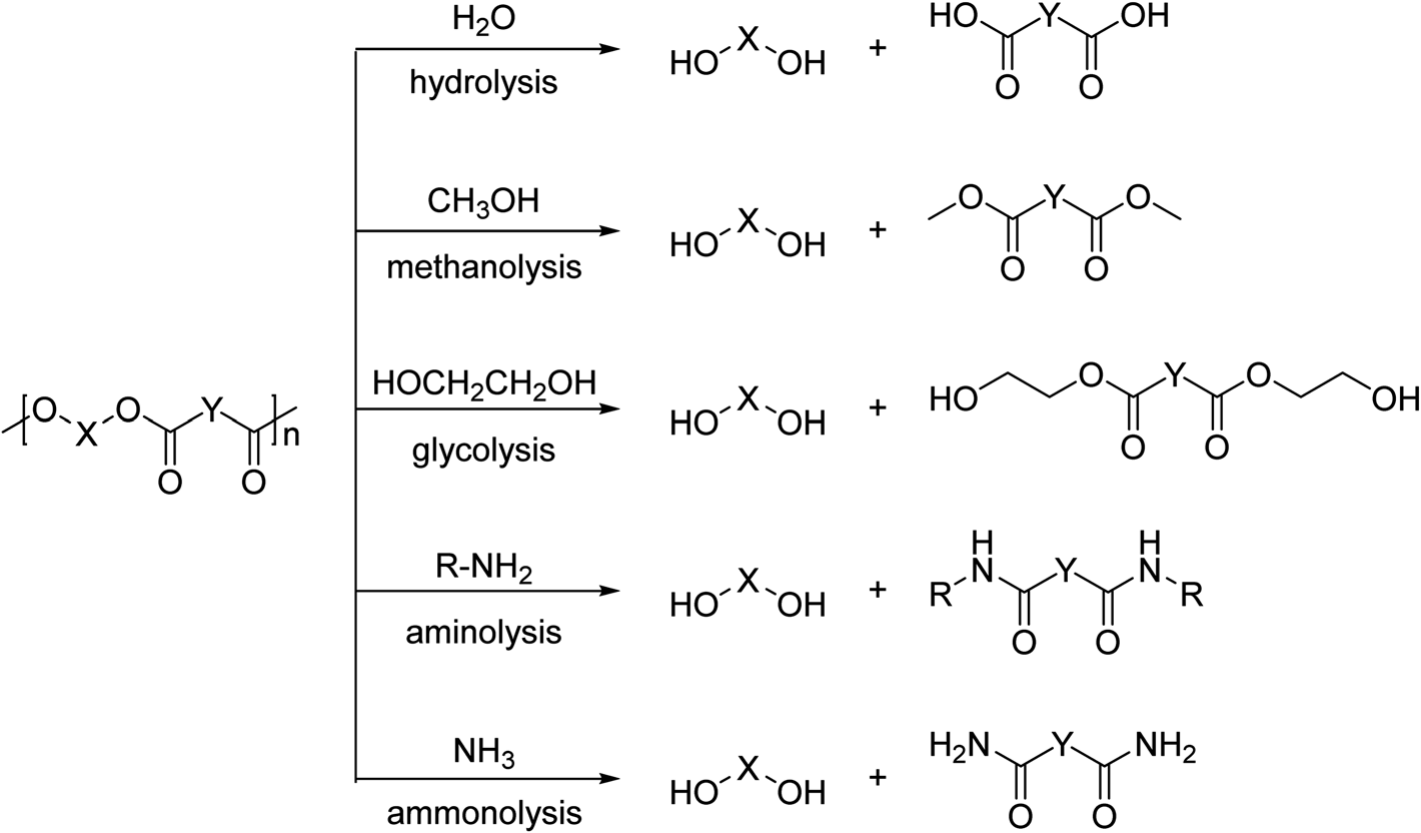
Schematic representation of solvolysis processes that are applied in the chemical degradation of polyesters. These processes are applicable to other polycondensates as well.

In contrast to these high temperature processes, solvolysis (or chemolysis) processes are operated at mild temperatures (<200 °C). Depolymerization in a solvent, often in combination with a catalyst, can only be applied to polycondensation polymers and chain cleavage yields the starting monomers or derivatives thereof. Solvolysis has the advantage that it yields a defined product that can be reprocessed into new polymers. Different solvents can be used to depolymerize polycondensation products (Fig. 1). When water is used as a solvent, the process is called hydrolysis. Most polycondensates are resistant to hydrolysis under normal conditions of use, and therefore hydrolysis requires elevated temperatures and/or pressure.^11^ Hydrolysis can be performed in acidic, neutral, or alkaline environments and yields the free acid monomer. However, hydrolysis processes are relatively slow as compared to other solvolysis processes due to the low nucleophilicity of water.^11–13^

Other solvents that can be used in the chemical depolymerisation of polycondensates are alcohols such as methanol or glycols such as ethylene glycol, diethylene glycol, or triethylene glycol. Depolymerization of polycondensates with aqueous amine solutions is called aminolysis and yields an amide as the product.^13,14^ Solvolysis in the presence of anhydrous ammonia is called ammonolysis.^11,14^ Analogous to hydrolysis, these processes require elevated temperature and/or pressure.^13,14^ The depolymerization mechanism is also different depending on the type of reagent and solvent used in the processes.^15–18^

## Enzymatic and microbial degradation

Another technique that has been gaining traction in the last decade as a potential method for the back-to-monomer recycling of polycondensation polymers is breakdown of the polymer chain using hydrolytic enzymes. Hydrolytic enzymes that break down naturally-occurring biopolymers have been studied for many decades, with examples including amylases, cellulases, xylanases, pectinases, chitinases and cutinases (see *e.g.* the CAZy database: http://www.cazy.org). Some of these enzymes are already employed in depolymerisation processes at industrial scale. For example, the breakdown of starch to glucose by amylases is a crucial step in the generation of bio-ethanol from first generation feedstocks, the production of which exceeded 100 billion liters in 2018.^19^ In the 2000s, it was discovered that some of these hydrolytic enzymes are also efficient catalysts for the depolymerisation of polycondensation polymers such as poly(ethylene terephthalate) (PET), which up to that point had been deemed recalcitrant towards enzymatic degradation.^20,21^ Since then, a large number of studies has been published on the enzymatic degradation of various types of polycondensation polymer and an enzymatic method for the degradation of PET is being commercialized by the French biotech company Carbios (http://www.carbios.fr).

Enzymatic hydrolysis relies on the catalytic activity of the involved enzymes (rather than on extreme pH values, pressures, or temperatures) to accelerate the hydrolytic depolymerisation of the polymer. This allows it to be performed under relatively mild reaction conditions^21–23^ (temperature < 100 °C, atmospheric pressure, neutral to mildly alkaline pH) compared to chemical hydrolysis methods, with potential benefits from the point of view of energy use and waste reduction. However, these advantages need to be balanced against the lower substrate concentrations and longer reaction times that enzymatic processes may necessitate. Another potential advantage of enzymatic depolymerisation processes lies in their selectivity. With chemical depolymerisation processes, it may be challenging to distinguish between different polycondensation polymers present in blends or composites. In contrast, enzymes may display inherent selectivity towards a single polymer, potentially enabling the selective depolymerisation of a single polycondensation polymer from a mixture while leaving the other components largely intact.^24^

The origin of enzymatic degradation lies in the breakdown of polymers in nature by microorganisms (bacteria or fungi) that excrete hydrolytic enzymes. Typically, these de-polymerization reactions evolved in order to enable the use of a polymer as carbon source. There are several examples in literature describing the metabolization or degradation of synthetic polyesters and other polycondensation products.^25^ Microbial degradation is likely limited to plastic degradation, *e.g.* for microplastics in water, but not suited for the back-to-monomer strategy for recycling polymers, as microbes that have evolved to degrade polymers will most probably also metabolize the resulting monomers to generate energy. However, isolation and characterization of micro-organisms capable of degrading synthetic polymers may be a highly useful way by which to identify novel polymer-degrading enzymes, as was recently demonstrated by the isolation of a novel PET-degrading enzyme from the bacterium *Ideonella sakaiensis* 201-F6.^22^

## (Bio)-chemical recycling of polyesters

**Scheme 2:**
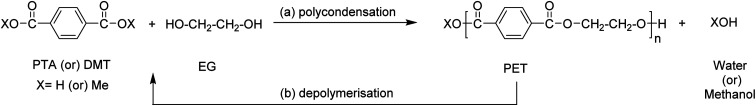
Synthesis of (a) PET *via* condensation reaction between PTA/DMT with EG; (b) depolymerisation of PET back to monomers.

Among a wide-range of commercially available polyesters, polyethylene terephthalate (PET) has received significant attention due to it being semi-crystalline or amorphous, as well as thermoplastic. These properties drive the use of this polymer in enormous application areas (amongst others, plastic bottles, (food) packaging, and fabrics) with a global production of 30.3 million tonnes in 2017.^26^ PET is typically produced by the melt-polycondensation of terephthalic acid (PTA) or dimethyl terephthalate (DMT) with ethylene glycol (EG) (Scheme 2a). The resulting ester bond offers the opportunity to be selectively broken under suitable reaction conditions, thereby enabling the degradation of the polymer to the original monomers.

The possibility to regain chemical building blocks from PET waste has motivated industry and academia to explore efficient processes to depolymerize PET and regenerate monomers (for example, PTA or DMT and glycols) or dimer/trimer mixtures such as bis(2-hydroxy ethyl)terephthalate (BHET) (Fig. 1). Monomers or dimer/trimers obtained can either fully or partially replace virgin monomers in the (re-)polymerisation into PET, and thus lower the need for fossil-based terephthalic acid.^27,28^

Solvolysis of PET has been successfully demonstrated using different solvents either in the presence or absence of a catalyst. Commonly employed solvents are water, amines, alcohol, and acids.^13,14^ Recently, combinations of the above mentioned methods such as glycolysis–methanolysis, methanolysis–hydrolysis, glycolysis–aminolysis were also successfully demonstrated for the depolymerisation of PET plastics.^13^ The following section will discuss the different solvolysis methods, and enzymatic methods, in more detail.

### Hydrolysis

PET is resistant to hydrolysis under normal processing and usage conditions. Hence high temperature and pressure are required, often in combination with harsh pH conditions, to break down the polymer chain into monomers (Scheme 2b).

An exception is the acid hydrolysis of PET involving concentrated sulfuric, phosphoric, or nitric acid, which does not require high temperature or pressure to facilitate the depolymerization.^11^ PET material treated with concentrated sulphuric acid at moderate temperature (90 °C) results in PTA and EG. However, severe corrosion of the reactors is a common problem encountered in this approach. Another disadvantage of acid hydrolysis lies in the costs for down-stream processing (DSP), since purification of monomers from concentrated sulphuric acid is costly. Hydrolysis in dilute sulphuric acid (<10 M) followed by subsequent separation of monomers by simple extraction or dialysis methods has been described in several patents.^29,30^ A smart way of separating PTA after hydrolysis is by the addition of an excess of potassium hydroxide (KOH) solution. While PET remains unaffected, PTA is transformed into dipotassium salts which can be easily separated by filtration.^31^ A wide-range of parameters like acid concentration, time, and temperature were investigated. Decrease of acid concentration and temperature caused extension of the reaction time.^32^ Though nitric acid supplied from an industrial side stream was advocated for PET hydrolysis, especially from a cost point of view, it is not commonly used. The EG obtained by PET depolymerisation in nitric acid is further oxidised to oxalic acid, due to the high oxidising ability of the acid used.^29^ This loss of EG makes the nitric acid process less favourable as a back-to-monomer strategy than hydrolysis in sulphuric acid, since only the PTA can be recycled.

Next to acid hydrolysis, alkaline hydrolysis has been shown to be effective by using either sodium hydroxide (NaOH) or potassium hydroxide (KOH) at various concentrations (4–20 wt%) yielding EG and a metal salt of PTA.^11^ The EG was separated using distillation and the PTA salt is further neutralized using a strong acid (for example H_2_SO_4_) to regenerate PTA. The residence time was shorter (3 h), but the temperature required for hydrolysis in alkaline conditions is higher (250 °C) than for to acid hydrolysis.^11^ The simultaneous alkaline hydrolysis of PET flakes followed by oxidation by oxygen resulted in a mixture of PTA salt and EG, where the latter is further converted into oxalate and CO_2_ similar to hydrolysis in nitric acid.^33^ The alkaline process is efficient at relatively low cost compared to acid hydrolysis, even when the PET materials are highly contaminated such as magnetic recording tapes, metallized PET films, or photographic films.^11^ Characterization of the alkaline hydrolysis under different reaction conditions revealed an increase in the PTA yield at increased reaction temperatures.^34^ The highest yield of 98% PTA was observed at short residence time (1.0 h) at 200 °C.^11,31^

Non-aqueous alkaline hydrolysis using ether solvents such as dioxane and THF in combination with an alcohol were also found to influence the depolymerisation of PET under basic conditions.^35^ The advantages of performing the depolymerisation reaction in a combination of solvents are significantly reduced operating temperatures (60–80 °C) compared to glycolysis method (>150 °C).^35^ Solid state depolymerisation was also investigated in an extruder. PET and solid NaOH pellets were mixed at 200 °C and the resulting EG was distilled out under reduced pressure. The sodium salt of PTA was left behind as a powder which enabled easy down-stream processing compared to the separation of PTA from an EG/water mixture.^11,36^ Besides non-aqueous strategies, various alternative aqueous solutions such as sodium *tert*-butoxide in *tert*-butanol, sodium isopropoxide in isopropyl alcohol, sodium methoxide in methanol, and sodium ethoxide in ethanol were investigated as solvents for PET depolymerisation. Among those, sodium ethoxide in ethanol was found to be most effective. Unfortunately the authors did not report the yield or selectivity of PTA production. PET depolymerisation in an aqueous ammonia solution at 200 °C results in the formation of the diammonium salt of PTA. The salt was subsequently acidified with concentrated sulphuric acid, which regenerated PTA in >99% purity.^37^

The costs of the processes mainly depend on the separation and purification of the resulting monomers. This particular issue has been addressed by introducing an additional step of oxidising impurities, thereby converting them into insoluble materials. PTA could be effectively separated from the insoluble residue in the reaction mixture.^11,38^ Phase transfer catalysts such as phenyltrimethylammonium chloride, hexadecyltrimethylammonium bromide, trioctylmethylammonium chloride, and trioctylmethylammonium bromide were employed in 5% aqueous sodium hydroxide solution to investigate the depolymerisation of post-consumer PET bottles. Interestingly, high yields of PTA up to 93% were obtained during this process.^39,40^

PET can be also hydrolysed at neutral conditions (pH) using water or steam in the presence of alkali metal acetates and at temperatures in the range of 200–300 °C.^11,41^ Despite various advantages like circumventing inorganic salts and corrosion free methodology (resulting from acid and base catalysed hydrolysis), mechanical impurities from the original polymer are left behind in the resulting PTA, which is considered of lower purity compared to PTA obtained from acid or alkaline hydrolysis. The continuous hydrolysis of PET in a twin-screw extruder resulted in oligomers containing 2–3 repeating units. The oligomers can be further depolymerised to monomeric PTA and EG.^36,42^ Hydrolysis of PET in acidic or basic conditions is possible, but the low nucleophilicity of water makes these process rather slow compared to other depolymerization agents, such as ethylene glycol.

### Glycolysis

**Scheme 3:**
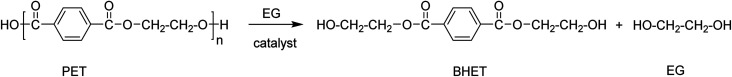
Glycolysis of PET using EG.

Glycolysis is a process of breaking PET polymer chains using an excess of glycols as a solvent at temperatures between 180–240 °C in the presence of trans-esterification catalysts.^43^ The resulting product is an α,ω,-dihydroxy compound of which the structure is dependent on the type of glycol used. For example, when EG is employed bis-hydroxyethyl terephthalate (BHET) is obtained as a product (Scheme 3).^44^

Commonly used solvents for the glycolysis of PET are EG, diethylene glycol, propylene glycol, dipropylene glycol, and triethylene glycol.^45^ Among these, EG is most widely used. Although glycols alone are capable of depolymerising PET at high temperature, the conversion yields are low and residence times are long. The presence of a transesterification catalyst such as zinc acetate (Zn(OAc)_2_) significantly accelerates the depolymerisation reaction. The catalysed glycolysis reaction has received significant attention by some chemical industries, due its high effectiveness, short residence time, and especially the huge market potential for the resulting α,ω-dihydroxy compounds in polyurethanes, polyesters, and epoxy resin synthesis.^11,36,46–49^ This prompted many researchers to investigate a wide-range of catalysts, especially metals salts, ionic liquids, and hydrotalcites.^14,45,50–54^

Wang *et al.* were the first to introduce ionic liquids as an alternative catalyst for PET depolymerization.^50^ Ionic liquids have advantages over metal and other catalysts as the process conditions are milder and, depending on the choice of ionic liquid, the process can be completely free of toxic compounds, including residual compounds. Different ionic liquids were investigated in detail by varying parameters such as temperature, time, and catalyst loadings. Among them, butyl-3-methylimidazolium bromide ([bmim] Br) showed the most effective performance resulting in 100% PET depolymerisation at a relatively low temperature and short reaction time of 180 °C and 8 h, respectively. The process yielded BHET as a sole product in high purity compared to the earlier results. The successful demonstration of the recovery and reusability of the ionic liquid used as catalyst is an additional advantage of this approach. Taking the whole strategy into consideration it was shown to result in a cost-effective recycling process.^55^ Iron-containing ionic liquids (*i.e.*, 1-butyl-3-methylimidazolium tetrachloroferrate ([bmim]FeCl_4_)) exhibited even higher catalytic activity at a lower temperature of 140 °C compared to ([bmim] Br) or ([bmim] Cl) at 180 °C. The high catalytic activity was attributed to the synergistic effect of its cation and anion. Metal containing ionic liquids such as copper acetate (Cu(OAc)2-[Bmim][OAc]) and zinc acetate (Zn(OAc)2-[Bmim][OAc]) and 1-butyl-3-methylimidazolium acetates are classified as neutral ionic liquids, which are easy to synthesise at low costs and display high efficiency for PET depolymerisation.^14,56^ Similar results were also achieved using poly-oxo metalates (POMs) K_6_SiW_11_MO_39_ (H_2_O) (M = Zn^2+^, Mn^2+^, Co^2+^, Ni^2+^).^57^

Deep eutectic solvents (DES) were also explored as a catalyst for PET depolymerization. Similar to ionic liquids, DES are easy to synthesise at very low cost, are very stable, non-toxic, and show good biological compatibility. 83% selectivity to BHET was obtained when using [urea/ZnCl_2_] DES with 100% PET conversion.^58^

Zeolites are advocated as an alternative heterogeneous system to heavy metal catalysts due to their acidity, large surface area, and pore size that can accommodate even high molecular weight PET chains. Mainly gamma (γ)- and beta (β) zeolites were investigated for PET depolymerisation. Both catalysts appeared to be effective in the depolymerisation of PET, however the yield of BHET is lower (65%) compared to that obtained with homogeneous catalysts (>80%)^59^ γ-Fe_2_O_3_, a super paramagnetic nanoparticle with a surface area larger than zeolites was employed for PET glycolysis depolymerisation.^60^

Metals salts (organometallics) play a prominent role as catalysts in PET depolymerisation studies as well. Different metal salts such as zinc acetate, sodium carbonate, sodium bicarbonate, sodium sulphate, and potassium sulphate were thoroughly investigated for their capabilities in PET glycolysis. The solvent thiodiglycol in combination with zinc acetate gave bis (2-(2-hydroxyethyl thio-ethyl terephthalate)) as a product.^61^ Metal oxides like ZnO, Co_3_O_4_, Mn_3_O_4_, and mixed metal oxides like ZnMn_2_O_4_, CoMn_2_O_4_, and ZnCo_2_O_4_ were also employed as catalysts for the depolymerisation of PET plastics.^14,62^ Among these, it is remarkable to mention that the monomer BHET can be obtained in up to 70% yield when using zinc acetate and EG as solvent. Although the operating temperature is slightly high at 196 °C, the PET to catalyst molar ratio is very high (380 : 1) in the presence of excess glycol, which is an important factor from a commercial process point of view.^14^ Although metal salts appeared to be suitable catalysts for PET depolymerisation, drawbacks with respect to toxicity of heavy metal salts do exist, creating a necessity for environmentally friendly catalysts. Sodium carbonate and sodium bicarbonate were found to efficiently depolymerise PET with comparable efficiency to zinc salts, giving similar yields at very low catalyst loadings.^54,63^ In addition to transparent PET plastics, combinations of metal salts and glycols are capable of depolymerizing highly coloured and multi-layered PET plastics in similar yields as well.^54,63^ Nevertheless, it has to be taken into account that for these specific products additional down-stream processing is required in order to remove coloured impurities from the obtained monomers.

Nano clay materials are eco-friendly, inexpensive, and abundantly available. The use of nano clay materials as catalysts in organic synthesis and polycondensation reactions has been receiving considerable attention lately.^12^ Hydrotalcite is composed of magnesium, aluminium, carbonate, and hydroxides. The layered double hydroxides are well known for their anion exchange properties. Dow Chemical Company has demonstrated the use of a hydrotalcite as an efficient catalyst for PET production.^12,64^ Going one-step further, Vivek Sharma *et al.*, studied the role of hydrotalcite as a depolymerisation catalyst especially for PET bottle chips.^45^ Hydrotalcite was capable of degrading PET bottle chips up to 98% in 10 minutes using dimethyl sulfoxide (DMSO) as a solvent at 190 °C. The products were mainly oligomers (tetramer), that required an additional step to hydrolyse the oligomers to DMT and EG using a strong base, for example sodium hydroxide. Nevertheless, the use of hydrotalcite has advantages such as a high reactivity, recyclability, and its non-toxicity.

In addition to the catalysed depolymerizations, glycolysis of PET was also conducted using microwave irradiation. The required activation energy (*E*_a_), evaluated using the Arrhenius equation, was found to be lower compared to the same process using conventional heating and at the same time the rate of the reaction was increased. Complete (100%) depolymerisation of PET was observed after 2 min in a microwave using DEG as solvent, whereas the complete depolymerisation required 8 h under conventional heating.^65^ While using EG as solvent, only 78% of the PET was converted to BHET after 35 minutes even in the presence of zinc acetate as a catalyst.^53^

It has to be mentioned that glycolysis does not necessarily have to result in monomers in order to obtain recyclable products: the glycolysates or partial glycolysis products obtained during PET depolymerisation can also find a number of other applications. Examples are the production of unsaturated polyesters by reaction of glycolysates with maleic anhydride and propylene glycol or with styrene.^11^ These unsaturated polyester products are used in manufacturing moulding products, gel coats, casting marble, bath fixtures *etc.*^11^ The polyester polyols or oligomers obtained during PET glycolysis can react with diphenyl methanediisocyanate (MDI) or toluene-2,4-diisocyanate (TDI) to product polyurethane products, for foam applications in particular.^66,67^ Furthermore, the oligomers or polyester polyols can be reacted with alkylene oxide for the production of polyurethane or polycyanurate foams possessing high fire resistance properties.^68^

### Alcoholysis

**Scheme 4:**
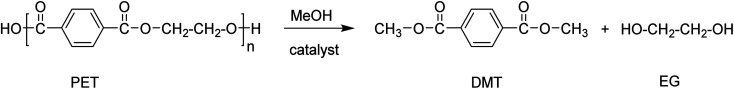
Alcoholysis using methanol (methanolysis) of PET resulting in DMT and EG.

Alcoholysis is a trans-esterification process that uses methanol or ethanol as a solvent, mostly performed at elevated pressure and temperatures ranging from 180–280 °C. The typical products resulting from the alcoholysis of PET using methanol are dimethyl terephthalate and ethylene glycol (Scheme 4).^69,70^

For example, PET can be depolymerised using superheated methanol vapours. EG and methanol are recovered by distillation and the former is re-used in fresh PET resin manufacture. Impure DMT is subjected to a hydrolysis/recrystallization process resulting in pure PTA, which can in principle subsequently be used in the manufacturing of fresh PET resin. An important prerequisite for this process is the use of organometallic catalysts or metal catalysts, such as zinc acetate, magnesium acetate, cobalt acetate, aluminum isopropoxide, or lead dioxide.^11,71^ When using such catalysts, it is important to deactivate the catalyst system after the depolymerisation to avoid further transesterification of DMT with EG in the presence of activated catalysts.

Recently, the efficiency of ionic liquids ([Bmim][BF_4_]) was demonstrated using supercritical ethanol resulting in diethyl terephthalate.^72,73^ The depolymerisation was carried out by subjecting PET to superheated methanol vapours which eventually break down the PET to DMT, mono methyl terephthalate, EG, and their simple derivatives. The process was shown to be successful using a wide range of different parameters that resulted in the formation of DMT in yields up to 97.7% at 310 °C.^74^ The process has been authenticated by several industries like Hoechst, DuPont, DOW Chemicals and Eastman.^11^ Mitsubishi heavy industries LTD (MHI) established a commercial process that involves the combination of methanolysis and hydrolysis reactions.^11^

### Aminolysis and ammonolysis

**Scheme 5:**
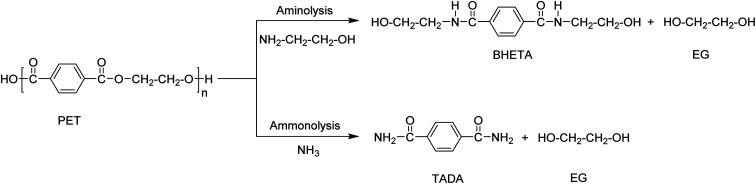
PET depolymerization using aminolysis and ammonolysis processes.

Ammonolysis and aminolysis yield terephthalic acid amide and amine derivatives, respectively (Scheme 5). These products cannot be used in the re-synthesis of polyesters, but have a potential in production of other classes of polymers such as poly(aryl ether sulfone amide).

The aminolysis process involves the use of amines such as methyl-, ethyl-, propyl-, butyl-, dimethyl-, or trimethylamine at temperatures between 20–100 °C in the presence of a catalyst such as zinc or lead acetate, acetic acid, or potassium sulfate. Bis (2-hydroxylethyl) terephthalamide (BHETA) is the main product when using ethanol amine (Scheme 5). BHETA has application potential in the production of novel polyurethanes, unsaturated polyester resins, bisoxazoline synthesis, and non-ionic polymeric surfactants.^75–78^ Although potential outlets have been explored using BHETA obtained *via* aminolysis, the process has not been applied at commercial scale. However, partial aminolysis is known for improving PET properties for applications such as in fibre manufacturing.^11,79^ The process was also used in combination with glycolysis. The depolymerisation of PET is first carried out using EG in a glycolysis process and then the products are treated with hydroxylamine resulting in terephthalohydrazide products which are used in the synthesis of water-reducible alkyd resins.^51,80–82^

PET depolymerization in the presence of ammonia at temperatures up to 180 °C is called ammonolysis. Ammonolysis of PET yielded up to 90% terephthaldiamide (TADA) as product with a purity >99% (Scheme 5).^83^ The reaction conditions included a temperature range of 120–180 °C at a pressure of 2 MPa. A low-temperature process using zinc acetate (0.05 wt%) as catalyst was also investigated and yielded 87% of TA amide at 70 °C. The obtained PTA amides were transformed into other classes of bulk chemicals such as *p*-xylenediamine or 1,4-bis(aminoethyl)cyclohexane.^11,14^

### Enzymatic hydrolysis

The enzymatic hydrolysis of polyesters, in particular of PET, has been a topic of substantial interest in recent years. Enzymatic processes for the hydrolysis of polyesters are advantageous due to the mild reactions conditions required, typically temperatures of below 100 °C and atmospheric pressure. Another advantage is that the substrate selectivity displayed by enzymes may enable their use to selectively depolymerise a particular polyester in a material consisting of multiple hydrolysable polymers, while leaving other polymers present unaffected. This strategy makes it potentially very useful for application in the recycling of articles composed of multiple polymers in *e.g.* blended textile materials or in plastic materials composed of multiple different polycondensation polymers (*e.g.* PET/PEF blends), which may become available in the market in the coming decades.

A number of enzymes have been described to be suitable for the depolymerisation of PET. The most prominent enzymes are cutinase-like polyester hydrolases. Cutinase-like hydrolases that have been studied for their PET-degrading activity include enzymes from the bacteria *Thermobifida fusca*, *Thermobifida cellulosilytica* and *Ideonella sakaiensis*, the fungi *Humicola insolens* and *Fusarium solani* and an enzyme identified from metagenomic analysis of leaf-branch compost (known as LC cutinase).^20–22,84–86^ Ronkvist *et al.* were the first to report that PET can be completely depolymerised by hydrolytic enzymes, achieving 97% weight loss of a 80 mg PET film after 96 h incubation with 0.6 mg (0.75 wt%) of (purified) *Humicola insolens* cutinase.^21^ Enzymatic depolymerisation of PET is typically carried out in an aqueous buffered solution at neutral to slightly alkaline pH as these are the reaction conditions under which the polyester hydrolases are most active.^21,22^ The depolymerisation is typically performed at temperatures ranging from approximately 30–80 °C. Reactions performed at the higher end of this temperature range have the advantage that they occur around the glass transition temperature of (semi-crystalline) PET, which is believed to increase the flexibility of the PET polymer chains and thus there accessibility for enzymatic hydrolysis. *H. insolens* and LC cutinases display relatively high activity at temperatures ≥ 50 °C, making these enzymes suitable for application in processes at relatively higher temperatures (50–80 °C).^21,22,87^

**Scheme 6:**
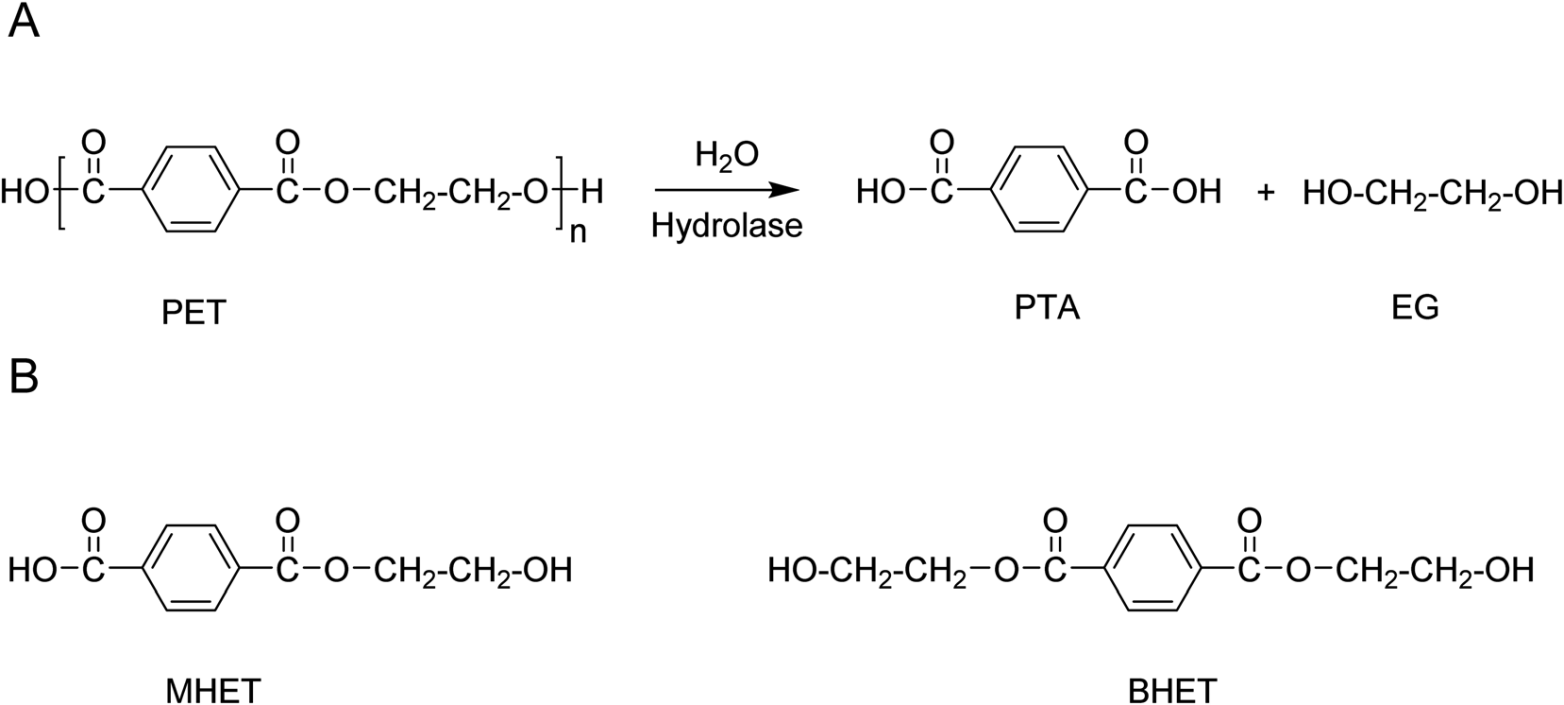
(A) Schematic representation of the enzymatic hydrolysis of PET to the monomers PTA and EG. (B) Structures of the aromatic monomers MHET and BHET that may be formed as side-products depending on the choice of enzyme and reaction conditions.

The products of hydrolase-catalysed depolymerisation of PET are typically the aromatic monomers, terephthalic acid (PTA), mono-2-hydroxyethyl terephthalic acid (MHET) and bis(2-hydroxyethyl) terephthalic acid (BHET) (Scheme 6). Typically, PET hydrolases are capable of fully hydrolysing PET, releasing PTA as the main product.^21,85^ However, depending on the enzyme and reaction conditions used, significant amounts of MHET may accumulate in the reaction mixture. For, *T. fusca* polyester hydrolase it has been shown that MHET is a relatively poor substrate for the enzyme and can in fact act as a competitive inhibitor of PET hydrolysis.^88^ Removal of MHET from the reaction mixture by the addition of a second enzyme with efficient MHET hydrolysing activity has been shown to increase the efficiency of polyester hydrolase-catalysed PET hydrolysis processes.^89,90^

Most studies concerning the depolymerisation of PET using polyester hydrolases have used pure PET materials with a relatively low crystallinity as the substrate for depolymerisation. Some recent advances have focussed on the development of processes for the depolymerisation of PET in more challenging materials, such as higher crystallinity PET fibres or materials containing PET as part of a multi-polymer blend. Recently, a chemo-enzymatic strategy for the hydrolysis of PET fibres to PTA was described.^91^ Here, neutral hydrolysis of the PET fibres at high temperature and pressure, which yielded a mixture of PTA and PET oligomers, was combined with a subsequent enzymatic depolymerisation of the obtained oligomers to yield PTA in high purity as a final product. Gamerith *et al.* reported that a cutinase from *T. cellulosilytica* can be used to hydrolyse PET in PET/PE and PET/PA blended polymeric materials, with enzymatic treatment leading to the release of PTA.^24^ However, the selectivity of the process for PET was not addressed, which would be in of particular interest for the PET/PA materials, as PA may conceivably also be depolymerised by a hydrolytic enzyme.

Enzymatic PET depolymerisation processes are in the process of being commercialised by the French biotech company Carbios (http://www.carbios.fr). Carbios was founded in 2011 and has developed a technology for the recycling of PET waste based on its depolymerisation by polyester hydrolases. In a recent report, researchers from Carbios and INSA Toulouse described a process for the production of PTA from post-consumer PET waste at a scale of 20 kg PET in a 150 L reactor.^23^ In this process, the post-consumer PET waste is first pre-treated by extrusion and micronisation to reduce the crystallinity and increase the surface area of the material. Then, the pre-treated PET waste is depolymerised by a variant of LC cutinase, optimised by enzyme engineering techniques to enhance its thermostability while maintaining depolymerisation activity similar to that of the wild-type enzyme. The reaction was performed in water at 20% (w/w) PET loading and 0.2% (w/w) enzyme/PET loading, with the temperature maintained at 72 °C and the pH maintained at 8 using an NaOH solution. Depolymerisation was allowed to proceed until it was 90% complete (after approximately 12 h in reactions at a scale of 20 g PET, not reported for larger scale reactions), after which the released PTA was purified by decolorization of the soluble fraction of the reaction mixture using a column of activated carbon, precipitation of the released PTA by acidification using H_2_SO_4_ and finally a crystallisation step to obtain PTA in >99.8% purity. Unfortunately, the final isolated yield of PTA was not reported. To support its commercialisation activities, Carbios holds various patent (applications) related to the polyester-hydrolase catalysed depolymerisation of PET and the corresponding enzymes.^92–101^ Carbios has claimed that its technology allows PET to be 97% depolymerised in a reaction time of 16 h and the technology has been demonstrated at a 1.000 L pilot scale.^23,102^ Carbios has announced the construction of a demonstration plant with an annual capacity of 50–100 kt that should be operational in 2021.^102,103^ In 2020 Carbios signed a joint development agreement with Novozymes, one of the market-leading enzyme suppliers, to supply PET-degrading enzymes for their recycling process, and in 2021 they announced an intention to construct a 40 kt per annum production unit located at the production facilities of PET-producer Equipolymers and to be operational in 2024.^103,104^

An overview of various chemical technologies for the depolymerisation of polycondensate polymers, in particular PET, is given in Table 1.

**Table 1:** An overview of selected reaction conditions for the technologies that have been developed for efficient depolymerization of PET back to monomers. Please note that the data reported in the table are chosen based on the highest yield of monomer obtained per technology

Technology developer	Starting material	Technology	Conditions						No. of steps^a^	Recovered		TRL^b^	Criteria^c^			Reference
			Solvent	Reagent	Catalyst	Time (h)	Temp. (°C)	Pressure (bar)		Monomer^d^	Yield (%)		*C* e	*P* f	*R* g	
Tohoku University – Japan	c-PET^h^	Acid hydrolysis	H_2_O	Sulfuric acid	—	1–6	150	ATM^i^	1	PTA^j^	100	3–4	±	+	±	^30^
Aristotle University – Greece	c-PET^h^	Base hydrolysis	H_2_O	Sodium hydroxide	—	1–2	200	ATM^i^	2	PTA^j^	98	3–4	±	+	±	^31^
Al-Mustansiriyah University – Iraq	c-PET^h^	Base hydrolysis	H_2_O	Sodium hydroxide	Tetrabutyl ammonium bromide	1–2	200	ATM^i^	2	PTA^j^	98	3–4	±	+	±	^105^
NIAIST-Japan	PET^k^	Neutral hydrolysis	H_2_O	H_2_O	—	0.5–1	420	480	1	PTA^j^	90	3–4	+	+	+	^106^
Zhejiang University of Technology – China	c-PET^h^	Neutral hydrolysis	H_2_O	H_2_O	Zn(OAc)_2_	0.5–1	220–300	32	1	PTA^j^	91	3–4	+	+	±	^107^
Imam Khomeini Int. University – Iran	c-PET^h^	Alcoholysis	Butanol	Butanol	KOH	N.D.^l^	100	ATM^i^	2	PTA^j^	96	3–4	±	+	±	^108^
Imam Khomeini Int. University – Iran	c-PET^h^	Alcoholysis	Pentanol	Pentanol	KOH	N.D.^l^	100	ATM^i^	2	PTA^j^	96	3–4	±	+	±	^108^
Kumamoto University – Japan	c-PET^h^	Methanolysis	Methanol	Methanol	—	0–1.5	300	147	1	DMT^m^	98	3–4	+	+	+	^109^
Chinese Academy of Sciences-China	c-PET^h^	Methanolysis	Methanol	Methanol	Zn(OAc)_2_	0.5–1	250–270	110	1	DMT^m^	95	3–4	+	+	±	^110^
UFRGS- Brazil	c-PET^h^	Glycolysis	Ethylene glycol	Ethylene glycol	Zn(OAc)_2_	2	196	ATM^i^	1	BHET^n^	83	3–4	+	+	±	^111^
Henan Normal University – China	c-PET^h^	Glycolysis	Ethylene glycol	Ethylene glycol	Zn(OAc)_2_	1–5	196	ATM^i^	1	BHET^n^	86	3–4	+	+	±	^112^
Institute of Process Engineering -China	c-PET^h^	Glycolysis	Ethylene glycol	Ethylene glycol	Bmim_2_[CoCl_4_]	1.5	175	ATM^i^	1	BHET^n^	96	3–4	+	+	±	^55^
Toulouse Biotechnology Institute (INSA), Carbios	PcW-PET^k^	Enzymatic degradation	H_2_O	H_2_O	Engineered polyester hydrolase (LCC cuntinase)	12	72	ATM	1	PTA^j^	90^o^	7–9	+	+	±	^100^
Polytechnic University New York	Gf-PET^p^	Enzymatic degradation	Buffer	H_2_O	Cutinase from *Humicola insolens*	96	70	ATM	1	N.D.	97^o^	3–4	±	N.D.	±	^21^
Institute of Chemical Technology-India	c-PET^h^	Aminolysis	Acetic acid	Ethanol amine	Sodium acetate	0–0.5	172	ATM^i^	1	BHETA^q^	91	3–4	±	+	−	^113^
Charan Singh University- India	c-PET^h^	Ammonolysis	—	NH_4_	Zn(OAc)_2_	360	RT^r^	ATM^i^	1	PTA diamide^s^	N.D.^l^	3–4	±	±	−	^114^

a Number of steps to obtain final monomer back.

b TRL: technology readiness levels according to EU definitions,^115^ estimated by the authors based on publicly available information.

c The symbols in the criteria section (estimated by the authors based on publicly available information) are defined as; +: high (or) attractive; −: low (or) less attractive; ±: average or not convincing.

d Type of monomer obtained from the process.

e Reaction conditions and possible scale-up options.

f Purity of monomer(s) obtained in the process.

g Recycling/reusing possibilities of solvents/reagents/catalysts used in the process.

h Commercial PET sample (Mn 30 kDa or higher).

i Atmospheric pressure.

j P-Terephthalic acid.

k Post-consumer PET waste fraction (mixed PET fraction), pre-treated by extrusion to obtain amorphous PET.

l Not described.

m Dimethyl terephthalate.

n Bis(2-hydroxyethyl) terephthalate.

o Degree of PET depolymerisation, isolated yield of PTA not reported.

p Amorphous PET film, commercially available from Goodfellow.

q Bis(2-hydroxy ethylene)terephthalamide.

r Room temperature.

s P-Terephthalamide.

## (Bio)-chemical recycling of polyamides

Polyamides (or nylons) are a class of polymers bearing amide (–CONH–) linkages between the monomeric units. Monomers such as (1) dicarboxylic acids and diamines, (2) ω-amino acids, and (3) lactams are typically used in the synthesis of polyamides.^116^ Due to the wide range of available monomers, polyamides can exhibit a broad range of properties suitable for many specific application areas.^116^ At their end-of-life, depolymerization of polyamides is mainly performed using hydrolysis and solvolysis techniques, which are detailed in the following sections.

### Hydrolysis

Since polyamides are built up from various types of monomers and thus have different backbones in the polymeric structure it is challenging to develop one unique strategy for their depolymerisation. Hence, the conditions can strongly differ based on the nature of the polymeric structures.

The hydrolysis of soluble nylon 11 (PA-11) in the presence of low molecular weight organic acids such as acetic acid, propanoic acid, and butanoic acid was investigated.^117^ Interestingly, it was found that the hydrolysis rate was inversely proportional to the acidity of the acid, *i.e.* the weaker the acid, the higher the hydrolysis rate. This phenomenon was explained by a higher affinity of the weaker acids for the polyamide structure. Experiments were performed at 100 and 120 °C. However, reaction times needed to reach an equilibrium were long (>20 days) and the average molecular weights of the hydrolysed products were still in the order of 15–30 kDa while the initial molecular weight was ∼70 kDa.

The strong base NaOH was found to be more effective as catalyst for the depolymerization of PA-6 into caprolactam.^118^ The maximum depolymerization degree (59.2%) for a PA-6 with an average molecular weight of 2.3 kDa was obtained after 300 min at 250 °C, and ∼4 MPa (600 psi) using a NaOH concentration of 10 wt% of the PA-6. In a later publication by the same authors, a full conversion of PA-6 (3.2 kDa) into caprolactam was achieved at 240 °C, ∼5 MPa (700 psi) and 60 min reaction time using the same NaOH concentration.^119^

Besides aliphatic polyamides, the depolymerization of aromatic polyamides (aramids) has also been investigated in detail. The hydrolysis of poly(*p*-phenylene terephthalamide) (Kevlar) fibres was studied in supercritical water, subcritical water, and subcritical water with NaOH.^120^ Supercritical water (*T* > 374 °C and *P* > 22.1 MPa) appeared not to be suitable for the chemical recycling of Kevlar fibres due to the incomplete polymer decomposition (decomposition efficiency < 100%) and the very low yields obtained for the monomers *p*-phenylene diamine (PPD, <35%) and terephthalic acid (PTA, <40%). The low yields of monomers were caused by their decomposition under the applied conditions. Milder reaction conditions (subcritical water) yielded only a decomposition efficiency of 17.5% after 90 min at 350 °C and 16.5 MPa. The addition of 3–5 mole equivalent of NaOH resulted in complete polymer decomposition after less than 5 minutes without monomer decomposition. The highest monomer yields of 98.9% for PPD and 95.3% for PTA were obtained with 4 mole equivalent of NaOH and 10 min reaction time. These quite extreme reaction conditions are not very suitable for an industrial process, therefore depolymerisation of Kevlar fibres was investigated at milder conditions. Decrease in the temperature and pressure to 250 °C and 4 MPa, respectively, and prolongation of the reaction time to 6 h resulted in comparable monomer yields (∼95%). Finally, it was shown that the monomers obtained could be easily recovered by extraction or precipitation techniques.^120^

### Solvolysis

Conventional glycolysis and amino-glycolysis processes for the chemical depolymerization of PA-6.6 (viscosity 1.5 dL g^−1^) have been described by Datta and co-workers.^121^ Ethylene glycol and a mixture of EG and triethylenetriamine (TETA) were used as decomposing agents. Typically, an excess of 3–6 mole equivalents of decomposing agent to PA-6.6 was used. The reactions were carried out at atmospheric pressure and elevated temperatures (190 °C) in the presence of 2 wt% diammonium hydrogen phosphate as catalyst. The reaction yielded substances with a molecular weight ranging from 90–250 g mol^−1^. In the case of glycolysis these substances were unreacted EG, hexamethylenediamine (HMDA) or oligomers containing β-hydroxyethylester end-groups. In the case of amino-glycolysis, oligomers containing amine end-groups originating from TETA were formed as well. The obtained substances were mixed in 10 wt% in a commercial polyol to synthesize successfully new poly(ester urethane)s.

Additionally, polyamides have been treated with supercritical secondary or tertiary alcohols. For example, the treatment of PA-6 with supercritical methanol yielded ω-hydroxyalkanoic acid derivatives.^122,123^ Reaction temperatures were between 300–370 °C with a pressure of 27 MPa. The treatment of PA-6 resulted in 6 different compounds (a mixture of caprolactams and ω-hydroxycapronic acids), with a total yield up to 82%, reached after a reaction time of ∼3 h. During the depolymerization, caprolactam was formed as the initial product, which was converted into mainly a 1 : 1 ratio of 6-hydroxycapronate and methyl 5-hexanoate.

The addition of an acid catalyst, such as glycolic acid, increased the yields of the more valuable ω-hydroxycapronic acids with a maximum yield of 85%.^124^ The technique was shown to be applicable for PA-6, PA-6.6, and PA-12. In addition, the reaction temperature could be lowered to 270 °C in the presence of the carboxylic acid catalyst.

### Enzymatic hydrolysis

A number of studies into the enzymatic hydrolysis of polyamide materials have been performed. A thermostable variant of an enzyme named nylon hydrolase (NylC_p2_) from *Arthrobacter* sp. has been shown to catalyse the hydrolysis of a nylon-6 powder, leading to the release of monomers and dimers into solution and the production of insoluble oligomers.^125^ The enzyme was also applied to the hydrolysis of thin layers of nylon-6, leading to at least 75% weight loss of the insoluble polymer.^126^ Nylon-66 was also degraded by the enzyme, although less efficiently than nylon-6. Further studies are required to establish whether this enzyme is suited for application in chemical recycling processes for polyamide materials.

A number of other hydrolytic enzymes, including proteases and cutinases, have been shown to display hydrolytic activity towards polyamide fibres.^127,128^ However, the focus of research to date has primarily been modification of the surface properties of textile by partial hydrolysis of the fibres and the ability of these enzymes to achieve full depolymerisation of polyamide materials has not been reported up till now.

## (Bio)-chemical recycling of polycarbonates

Polycarbonates (PCs) are a class of polymers that have an unique combination of properties such as high toughness, transparency, and high heat distortion resistance. The term PC is often used for the economically most relevant PC, bisphenol A polycarbonate (BPA-PC). The major application areas of PCs are electronics, the automotive sector, and the building and construction sector. To improve their properties, such as toughness or solvent resistance, PCs are also blended with a broad range of other polymers such as acrylonitrile-butadiene-styrene (ABS), poly(butylene terephthalate) (PBT), or poly(ethylene terephthalate) (PET).^129^ It is estimated that more than 20% of the produced PC is used in blends. The increasing market share and production of PCs create a need for sustainable recycling of these polymers.

### Hydrolysis

**Scheme 7:**
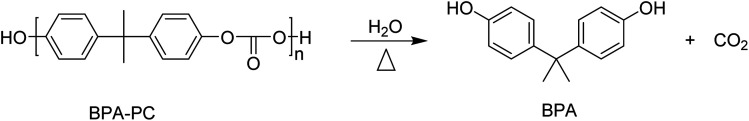
Schematic representation of BPA-PC hydrolysis into BPA and CO_2_.

The hydrolysis of BPA-PC results in the formation of bisphenol A (BPA) and CO_2_ (Scheme 7).

**Scheme 8:**
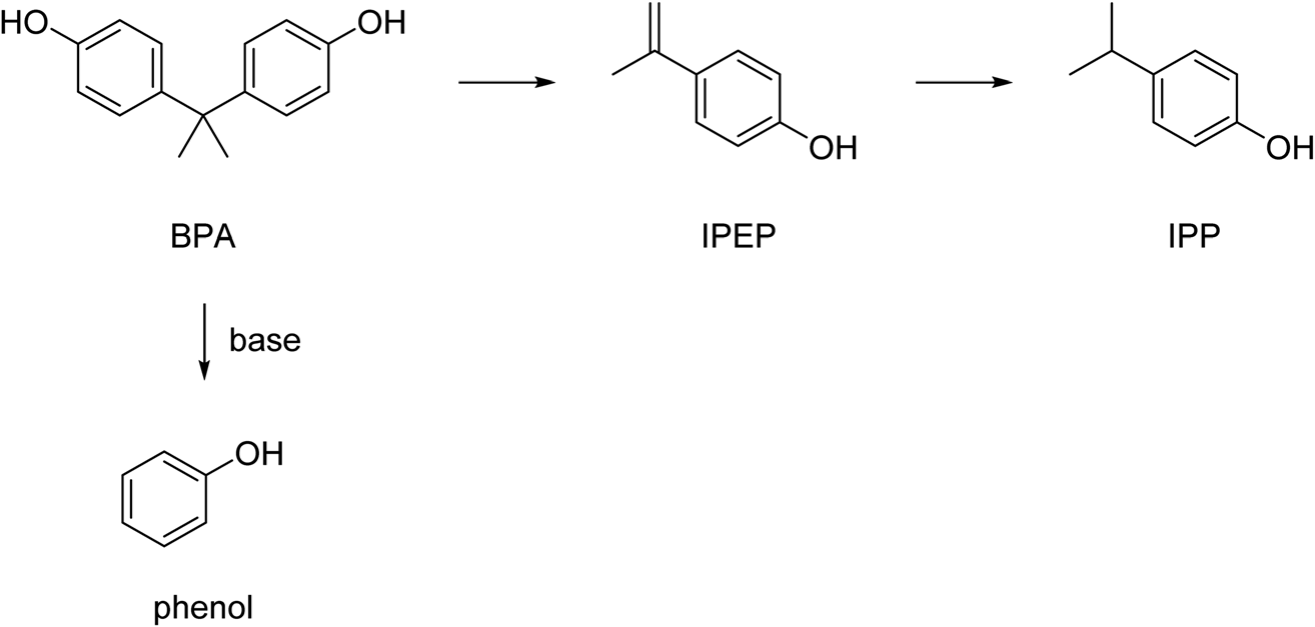
Schematic representation of possible secondary reactions of BPA in water.

However, under the applied hydrolysis conditions secondary reactions can occur that further convert the formed BPA. The depolymerization of BPA-PC and the products formed has, for example, been studied in sub- and supercritical water.^130^ The highest yield of decomposition products phenol, bisphenol A, *p*-isopropenylphenol (IPEP), and *p*-isopropylphenol (IPP) (Scheme 8) was 88.9% and obtained at relatively high temperatures (430 °C) at a residence time of 1.0 h. Lower temperatures resulted in significantly lower yields of the decomposition products and no BPA-PC depolymerization was observed at temperatures below 230 °C. The addition of salt or acetic acid in the near supercritical region (250–300 °C) was found not to have a significant effect on the yield either. The addition of sodium carbonate, however, accelerated the decomposition, and made phenol the main reaction product due to the instability of BPA under these alkaline conditions.^130^

The use of an ionic liquid catalyst enables the decrease of reaction temperatures. One-butyl-3-methylimidazolium acetate was identified as an effective catalyst.^131^ Nearly full conversion of BPA-PC was obtained at a relatively low temperature (140 °C) within 3.0 h. The yield of BPA was over 96%. The ionic liquid could be reused up to 6 times without affecting the BPA-PC conversion and BPA yield. Although high yields were obtained, given the cost of ionic liquids, their use is regarded as too expensive compared to conventional base catalysts, such as acid or base catalysts.

Iannone, *et al.*,^132^ used a bifunctional acid/base catalyst (zinc oxide and tetrabutylammonium salts) and various nucleophiles to depolymerize BPA-PC, avoiding the use of harsh conditions. By tuning the nucleophile, a wide range of linear or cyclic carbonates or urea could be obtained in excellent yields (>97%).

Most studies use the neat PC polymer to test the depolymerisation, while the presence of additives can have a strong effect on the depolymerization. The effect of a flame retardant (decabromodiphenyl ether) and plasticizer (di-*n*-octyl phthalate) on the depolymerisation of BPA-PC in subcritical water was tested by Huang and co-workers.^133^ The presence of the flame retardant accelerated the hydrolysis while the presence of the plasticizer had an inhibiting effect. Both additives reduced the yield of BPA and increased the phenol yield.

BPA-PC can be hydrolysed in steam instead of water in a pyrolysis-like process. Steam requires less water and hence the process requires less energy. BPA-PC hydrolysis in steam with magnesium or calcium oxides or hydroxides at 300 °C, resulted in the formation of BPA as the main product with a maximum yield of 78%.^134^ At 500 °C, BPA is cleaved into phenol and IPEP. The best results were obtained in a two-step process where BPA-PC is hydrolysed at 300 °C, and the resulting BPA is cleaved at 500 °C resulting in a phenol yield of 84% and an IPEP yield of 64%. The differences in hydroxide or oxide form of the catalysts were found to be negligible, however, magnesium catalysts performed better than calcium catalysts with respect to decomposition product yields. The use of a magnesium catalyst has the additional advantage that magnesium hydroxide is already used as fire retardant in many PCs, and therefore the addition of additional catalyst can be minimized. However, the MgO catalyst deactivates over time due to the deposition of organic residue.^134^ Steam in combination with a high pressure was also applied to hydrolyse BPA-PC.^135^ At 300 °C and 7.9 MPa, a maximum BPA yield of around 80% was achieved after 5 min, whereas in liquid water (300 °C, 12.2 MPa) PC did not show depolymerization after 50 min.

Microwave heating has the advantage that the heat simultaneously heats the bulk of a material in a fast manner. The hydrolysis of BPA-PC in a microwave was studied under alkaline conditions in the presence of a phase transfer catalyst (PTC).^136,137^ The PTC was found to be essential; in the absence of PTC no depolymerisation was observed. The highest BPA-PC conversion obtained was 80%, and the BPA monomer was obtained in quantitative yield. Optimal reaction conditions were 160 °C, and a reaction time of 10 min with 10% NaOH and a reaction time of 40 min with 5% NaOH.

Although BPA-PC is the most studied PC, aliphatic PCs have an important market potential as well. The hydrolytic degradation of the aliphatic PC poly(propylene carbonate) in THF solutions containing 10 wt% acids or base at 30 °C was studied. No drop in viscosity, and thus molecular weight, was observed in the pH-range 5.0–9.0 up to 20 days. At acidic conditions, a moderate drop in viscosity was observed. A more strong and rapid drop in viscosity was observed for strong alkaline conditions (pH 13). After 20 days at pH 13 the weight average molecular weight had dropped from an initial value of 673 kg mol^−1^ to 43 kg mol^−1^. Although the polymer had degraded to some extent, the applied conditions were too mild to allow complete depolymerization back to its monomers.^138^

### Solvolysis

**Scheme 9:**
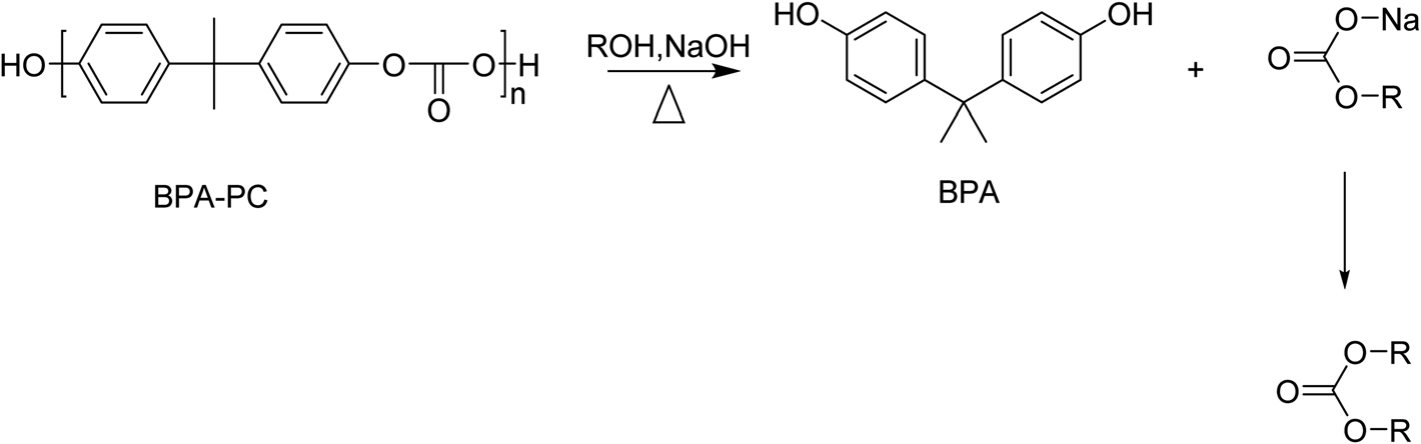
Schematic representation of alcoholysis of BPC-PC by an alcohol (ROH) in the presence of NaOH.

Solvolysis of BPA-PC using small alcohols such as methanol or ethanol results in the formation of BPA and a carbonate (Scheme 9).

The alkali-catalysed depolymerization of BPA-PC in a semi-continuous lab-plant using methanol at near critical conditions was studied. Optimal reaction conditions (80–90% kg BPA/kg PC and 35% kg dimethyl carbonate/kg PC) were found at 120–140 °C, 10 MPa, and 1.5–2 kg m^−3^ NaOH in pure methanol. As well as lowering the pressure, the presence of water resulted in the formation of by-products, such as phenol, and lowered the yield of the desired products.^139^

Solvolysis can also be performed with addition of ionic liquids. Methanolysis of BPA-PC in the presence of the ionic liquid 1-*n*-butyl-3-methylimidazolium chloride resulted in full PC conversion with BPA and dimethyl carbonate (DMC) yields over 95% at 105 °C for 2.5 h. The ionic liquid could be reused 8 times without affecting its performance significantly. As mentioned already before, ionic liquids are, however, typically more expensive as compared to the simple base catalysts often applied.^140^

Depolymerization of BPA-PC in supercritical ethanol resulted in the formation of BPA and diethyl carbonate (DEC). Ethanol reaches it supercritical state at milder reaction conditions than methanol or water. Only 7.5% BPA-PC conversion was observed in the subcritical region (at 240 °C), whereas complete degradation could be obtained in 30 min in the supercritical region (290 °C, 8.5 MPa). BPA and DEC yields in supercritical ethanol were 90% and 89%, respectively.^141^

The organocatalyst 1,8-diazabicyclo[5.4.0]undec-7-ene (DBU) was used in the alcoholysis of BPA-PC. DBU was found to be more active than NaOH and other organocatalysts.^142^ Selective conversion of BPA-PC into BPA and DMC in methanol was observed in about 30 min at 100 °C and a DBU load of 10 mol%. In ethanol, BPA and DEC could be obtained in quantitative yields as well, however, it required longer reaction times (3.25 h *versus* 30 min). Mixtures of methanol and ethanol resulted in a mixture of DMC, DEC, and the mixed carbonate ethyl methyl carbonate. Mixed carbonates are potential precursors for the synthesis of unsymmetrical ethers ROR′ by decarboxylation of the carbonate.

**Scheme 10:**
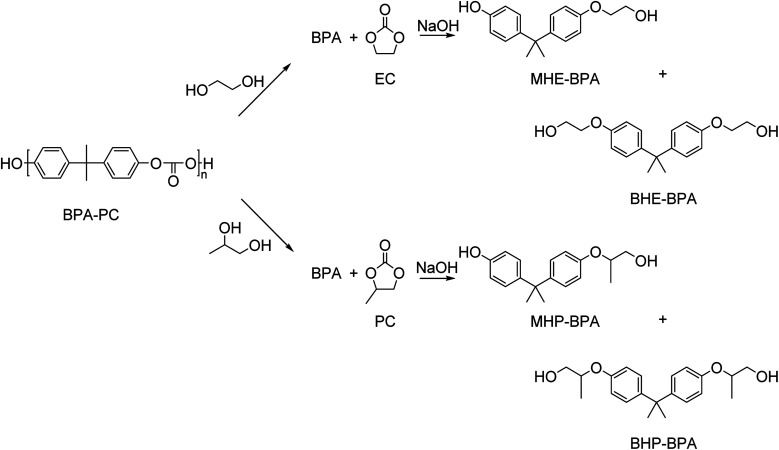
Glycolysis of BPA-PC by ethylene glycol or 1,2-propanediol yields in the formation of BPA and ethylene carbonate (EC) or propylene carbonate (PC), respectively. Subsequently, the reaction of BPA with a carbonate in the presence of an inorganic base such as NaOH yields the corresponding mono- and bis(hydroxyalkyl)ethers. Note that for MHP-BPA and BPH-BPA multiple stereoisomers exist (not drawn).

Besides small mono-alcohols such as methanol and ethanol, bifunctional alcohols such as ethylene glycol, 1,2-propanediol, and glycerol have been used in the depolymerization of BPA-PC. Typically, these reactions yield a mixture of BPA, mono-, and bis(hydroxyalkyl)ethers (Scheme 10).

BPA-PC was glycolyzed with full conversion by an excess of ethylene glycol in the presence of a catalytic amount of NaOH at 180 °C for 10 min. Three products were observed: BPA (42%), monohydroxyethyl ether of BPA (MHE-BPA, 42%), and bishydroxyethyl ether of BPA (BHE-BPA, 11%). When 1.6 mol equivalence of ethylene carbonate was added, the bishydroxyethyl ether of BPA could be produced quantitatively at the same reaction conditions.^143^ Ethylene carbonate, however, is currently not an industrial commodity.

In a similar study, BPA-PC was glycolyzed with ethylene glycol (EG) or propylene glycol (PG) with a catalytic amount of sodium carbonate at 180 °C under atmospheric pressure.^144^ When EG was used, BPA was obtained in 28% yield, MHE-BPA in 40% and BHE-BPA in 25%. When PG was used, BPA was obtained in 26% yield, monohydroxypropyl ether of BPA (MHP-BPA) in 53%, and bishydroxypropyl ether of BPA (BHP-BPA) in 21%. Treatment with an excess of the inexpensive urea with a catalytic amount of zinc oxide at 180 °C resulted in conversion to BHE-BPA and BHP-BPA. The formed bisalkoxylated diols of BPA were subsequently successfully used as a monomer in the synthesis of polyurethanes.

Interestingly, when BPA-PC was depolymerized with 1,2-propanediol or glycerol in the presence of DBU the reaction yielded BPA and the corresponding cyclic carbonate with very high selectivity.^145^ Under solventless conditions (using polyol as both the reagent and reaction medium), yields of the cyclic carbonate (up to 87%) were obtained at 180 °C. Addition of THF, a co-solvent in which BPA-PC is soluble, resulted in nearly quantitative carbonate yields at mild reaction conditions (near room temperature and a stoichiometric amount of polyol).

Hidaka and co-workers^146^ used the carbohydrates glycerol and glucose as polyols in the chemical depolymerisation of BPA-PC with the aim to generate value from the carbonate unit. The authors call this process quasi-solvolysis since a stoichiometric amount of reagent was used in combination with an inert solvent. By using glycerol and KOH in dioxane, a reaction temperature of 100 °C and a reaction time of 25–60 min, a hydroxymethyldioxolane yield of 97% and a BPA yield of 100% could be obtained. Treatment of BPA-PC with glucose in NaOH and pyridine at 110 °C for 110 min resulted in a glucose dicarbonate yield of 40% and a BPA yield of 42%. Thus, glycerol is more effective in the coproduction of both BPA and organic carbonates.

### Aminolysis

**Scheme 11:**
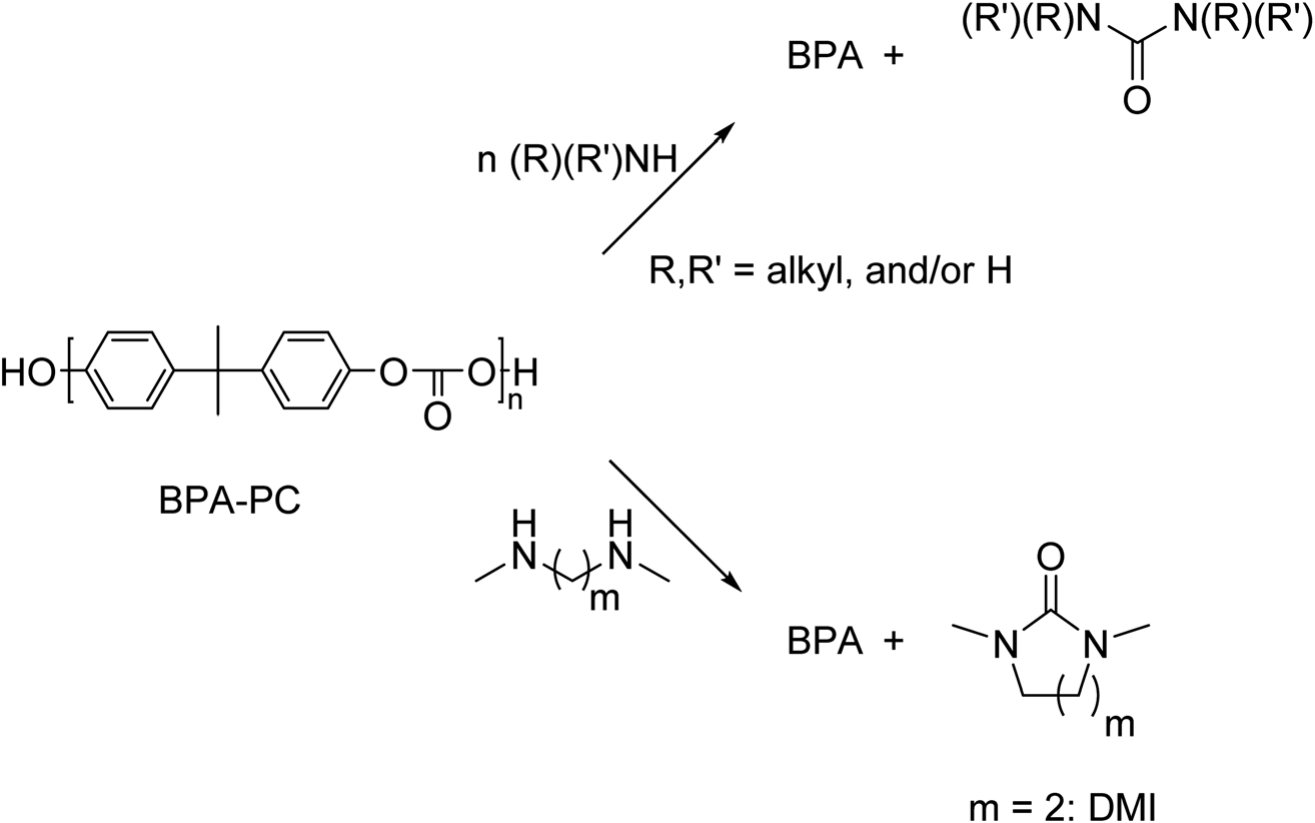
Schematic representation of aminolysis of BPA-PC by mono- and bifunctional amines.

Aminolysis with mono-functional primary amines results in the formation of BPA and 1,3-disubstituted ureas (Scheme 11). These urea are used in many applications, such as dyes, antioxidants, corrosion inhibitors, and as used as intermediate for the preparation of pharmaceuticals and agricultural chemicals.^147^

Aminolysis of BPA-PC by monofunctional primary amines was performed at 80 °C.^148^ This method did not require the addition of a catalyst, but ethanol or isopropanol was necessary as an additional solvent depending on the amine chosen (various derivatives of benzylamine), yields of the corresponding urea derivatives varied between 28–81%. In addition, a selection of alkyl amines showed yields between 50–81%. Arylamines (various derivatives of aniline) did not react with the BPA-PC, probably due to the low nucleophilicity of the aryl amines.

In contrast to the primary amines, aminolysis of BPA-PC with the monoamines dimethyl amine and diethyl amine resulted in very low yields.^149^ Aminolysis with bifunctional amines was also described and resulted in the formation of BPA and cyclic carbonates as degradation products. The BPA-PC degradation with diamines proceeded smoothly. Treatment of BPA-PC with *N*,*N*′-dimethyl-1,2-diaminoethane in dioxane with a catalytic amount of base for 30 min at 100 °C yielded 1,3-dimethyl-2-imidazolidinone (DMI, 94%) and BPA (95%). Slightly lower yields of 93% and 91%, respectively, were obtained when DMI was used as solvent. However, in DMI there was no need for an additional solvent.^149^

**Scheme 12:**
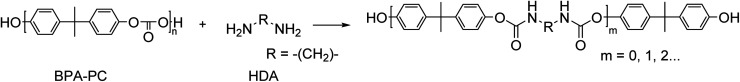
Schematic representation of the formation of hydroxyl terminated biscarbamates in the absence of any catalyst and at mild reaction conditions.

In general, most depolymerisation processes require high temperatures, and the use of a catalyst and/or co-solvent. Moreover, depolymerisation reactions often result in multiple products that have to be separated which increases the cost of down-stream processing and loss of material. A one-pot process that converts the depolymerization products into new molecules will reduce the costs and material losses. Wu and co-workers^150^ report on an aminolysis process at mild conditions of which the resulting products could subsequently converted into polyurethanes in a one-pot reaction. The authors treated BPA-PC with aliphatic diamines to obtain hydroxyl terminated biscarbamates in the absence of any catalyst and at mild temperatures (<80 °C) (Scheme 12). It was found that the choice of solvent was the key factor in achieving high yields without the formation of urea by-products. The formed pre-polymers were reacted with diisocyanates to form polyurethanes without any prior isolation or purification of the depolymerization products.

### Enzymatic

Relatively little is known about the enzymatic degradation of polycarbonates. In the 1990s, Suyama and Tokiwa reported on the degradation of the aliphatic polycarbonate poly(tetramethylene carbonate) using a lipoprotein lipase from *Pseudomonas* sp. The polymer could be completely degraded in an aqueous suspension, yielding 1,4-butanediol and di(4-hydroxybutyl)carbonate as the reaction products.^151^ It may be an interesting line of future research to explore whether hydrolytic enzymes that are known to degrade other polymer classes can be employed in the degradation of polycarbonates.

## (Bio)-chemical recycling of polyurethanes

Polyurethanes (PUs) are produced *via* polycondensation of polyols and isocyanates. A wide diversity of polyols and isocyanates have been applied, resulting in a broad range of PU materials. PUs can be classified in two main categories: rigid, or flexible foams and coatings, adhesives, sealants, and elastomers. Nowadays, glycolysis is the most-used industrial chemical recycling method for polyurethane recycling.^152,153^ PU scrap (preferably rigid PU foam) is heated to 180–220 °C in the presence of a glycol (typically a combination of diethylene glycol (DEG) with diethanol amine as co-reagent) and a catalyst.^154^ At lower temperatures the catalyst activity is too low, whereas higher temperatures cause undesired side reactions towards amines. PU glycolysis is sensitive to the presence of water due to the formation of diamines as PU degradation products. These diamines can potentially form a risk for health and the environment and have to be removed by distillation using batch-wise washing with clean DEG.^155^

### Hydrolysis

The degradation of PU in the presence of water results in the formation of diols and diisocyanates, of which the latter further reacts with water to form primary diamines. Hydrolysis of PU foam at 200 °C and 16 bar (230 psi) resulted in 85% conversion to polyamines and 90% to polyols 90% based on the amine and hydroxyl value, respectively.^156,157^

Motokucho and co-workers^158,159^ used pressurized CO_2_ in water to hydrolyse PU synthesized from 1,4-butanediol and either 1,6-hexamethylene diisocyanate, isophorone diisocyanate, or 4,4′-diphenyl methane diisocyanate. The depolymerization temperature was varied between 90–190 °C, the time up to 52 h, and the CO_2_ pressure from 2–16 MPa. The degree of hydrolysis obtained was >90% for all PUs. The rate of hydrolysis was highest for 1,6-hexamethylene diisocyanate, followed by isophorone diisocyanate and 4,4′-diphenyl methane diisocyanate. This difference in hydrolysis rate was probably caused by the hydrophilicity around the urethane linkage in the polymer terminal moiety, which is highest for 1,6-hexamethylene diisocyanate.

### Solvolysis

Flexible PU foams based on polyester and polyether polyols and toluene diisocyanate can be treated with triethyl phosphate at 190 °C to yield a mixture of phosphorous oligo-urethanes. The products formed have the potential to be used in the synthesis of new polymers with reduced flammability, improved adhesion, or improved resistance against UV-irradiation.^160^ Diethylene glycol was also used as solvent in combination with potassium carbonate as catalyst to dissolve flexible PU foam at a reaction temperature of 220 °C. The resulting product was a polyol-containing liquid, which was purified by distillation. After distillation, the total yield of these polyol-containing liquid was in the range of 25–60%.^155^

Simón, *et al.*,^161^ published a detailed study of the glycolysis of flexible PU foams containing polymeric polyols. The authors showed that these PU foams can be depolymerized using similar reaction conditions to those used for conventional PU foams containing flexible polyether polyols. In addition, the study showed that the recycled polyol could be used in the production of new PU foams.

Datta, *et al.*,^152^ used distilled glycerine (99.5%) and crude glycerine (80%) obtained as side stream from biodiesel production in the glycolysis of elastomeric PU. Potassium acetate was used as catalyst and the temperature was kept at 225–230 °C. The type of glycerine did not have significant influence on the chemical composition of the glycolysis products and the resulting polyols could be used in the production of new elastomeric PU.

The dissolution of flexible integral skin PU foam in a mixture of DEG and diethanol amine was found to be most optimal at temperatures 200–205 °C. At temperatures below 170 °C the foam did not depolymerize, whereas temperatures above 210 °C resulted in PU foam depolymerization and solvent evaporation.^162^ The recycled polyol was blended with virgin polyol up to 60 wt% to create recycled PU foams.

Microwave irradiation has been used to dissolve flexible PU foam.^163^ In comparison to conventional heating methods, short heating times could be achieved (20–30 times shorter) to completely dissolve the PU foam. Again the alkaline metal hydroxides were used as catalyst, and glycerine was used as glycol, however no yields were given.

Various reaction conditions and methods were investigated for the dissolution of rigid PU foams. The alkaline metal hydroxides NaOH and KOH were found to be the most effective catalysts when applied equal weight concentration.^162,164^ Additionally, glycols were applied with varied the number of carbon atoms. The dissolution time of the PU in the presence of 0.2 wt% KOH at 180 °C was found to be strongly dependent on the molecular weight of the glycol with the shortest dissolution times for tetraethylene glycol and dipropylene glycol.

### Enzymatic hydrolysis

Due to their complex and heterogeneous structures, polyurethanes are interesting candidates for recycling processes involving selective enzymatic hydrolysis. Selectivity displayed by hydrolytic enzymes may confer them with the ability to selectively hydrolyse a specific segment of a polyurethane material, *e.g.* selectively degrading the polyol segment while leaving the urethane segment intact or *vice versa*.

A number of enzymes that can degrade polyurethane materials have been described. In the 1990s, a number of hydrolytic enzymes were identified in the fungus *Curvularia senegalensis* and various bacterial *Pseudomonas* species that were capable of breaking down the water dispersible poly (ester urethane) Impranil.^165–168^ The degradative activity of these enzymes was primarily characterised by studying their ability to clear Impranil from agar plates and the reaction products formed from their activity were not characterised. An esterase from *Comamonas acidovorans* was shown to degrade the polyester segment of a poly(ester urethane) cube synthesised from poly(diethylene glycol adipate) and 3,4-toluene diisocyanate, leading to the release of diethylene glycol and adipic acid.^169^ A number of polyester hydrolases known for their PET-degrading activity have also been shown to be active towards Impranil and the poly (ester urethanes) Elastollan B85A-10 and C85A-10.^170^ FTIR analysis indicated that these polyester hydrolases cleave the polyester bonds of the polyol segments of the polymer.

Gamerith *et al.* reported on the degradation of pellets of the poly (ester urethane)s PU 1080 and PU 1050 by an amidase from *Nocardia farcinica*.^171^ This led to the release of various PU oligomers and the monomeric compound 4,4′-methylenedianiline, indicating that this amidase can cleave both ester and urethane bonds. Recently, an esterase and an amidase were combined in a two-enzyme synergistic process for the degradation of a polycaprolactone and 4,4′-methylene diisocyanate-based thermoplastic polyurethane, releasing monomers of both the soft (6-hydroxycaproic acid) and hard (4,4′-methylenedianiline) segments into the soluble fraction.^172^ These degradation processes do not regenerate the diisocyanate monomers employed in polyurethane synthesis, but the obtained diamines can serve as precursors for their synthesis.

## Commercial status of chemical and enzymatic recycling

There is only a limited number of companies that are currently recycling PET chemically at industrial scale or in a pilot phase (Table 2). The main reasons for the limited number of operating plants are the presence of impurities or contaminants (inks, paper, glue) in PET waste, the presence of co-monomers in PET, the high process costs, and the lack of a constant quality and amount of feedstock. The chemical recycling processes that are mentioned for PET could also be applied to other polyesters. However, the availability of feedstock is even lower for these polymers.

**Table 2:** Overview of chemical companies involved in the recycling of PET polymer

Company	Type of PET plastic	Technology	Catalyst/reagent/medium	Monomer/product	Development stage	Processing capacity	Reference
Gr3n (DEMETO) -SW	Bottle grades, packaging, textile	Alkaline hydrolysis	Microwave, base	PTA^a^	Pilot plant	60 kg h^−1^	^173–175^
Eastman	Bottle grade, scrap from various products	Glycolysis/methanolysis	N.I.A.^b^	BHET^c^/DMT^d^	Pilot plant	N.I.A.^b^	^15^
Loop	Low value material, fibre, carpet, scraps, ocean debris	Methanolysis	N.I.A.^b^	DMT^d^	Early commercial	N.I.A.^b^	^173^ and ^176^
IBM (VolCat) – USA	Bottle grade, packaging, scrap from various products	Glycolysis	DBU^e^ (IBM)	BHET^c^	Early stage towards pilot plant	N.I.A.^b^	^15^
Garbo (ChemPET) – IT	Bottle grade, multi-layer packages, fibres, textiles	Glycolysis	N.I.A.^b^	BHET^c^	Pilot plant	1000 t per year	^173^ and ^177^
Ioniqa Technologies – NL	Bottle grades, textiles, multi-layer packages	Glycolysis	Magnetite, [Bmim][FeCl_4_]	BHET^c^	Commercial pilot plant	10 kt per year	^173^ and ^178^
Jeplan (BRING)	Bottle grade, fibre, textiles	Glycolysis	Sodium, methylate, carbon^f^	BHET^c^	Pilot plant	20–25 kt per year	^173^
PerPETual – UK/IN	Bottle grades	Glycolysis	N.I.A.^b^	Low MW oligomers	Commercial plant	∼2 million plastic bottles per day	^173^ and ^179^
Resinate Materials Group (Recyolysis)	Bottle grade, scrap from various products, PETG	Glycolysis	N.I.A.^b^	Polyester polyol^g^	Pilot plant	200 metric ton and 4500 metric ton	^173^ and ^180^
Carbios – FR	Bottle grades, textile, fibres	Enzymatic	Engineered PET-depolymerase	PTA^a^	Pilot plant under construction	N.I.A.^b^	^23^ and ^102^
BP (Infinia)	Bottle, unrecyclable scrap from various products	Hydrolysis	N.I.A.^b^	PTA^a^	Early stage of Pilot plant	N.I.A.^b^	^181^
Poseidon Plastics	Scrap from various products	Glycolysis	N.I.A.^b^	BHET^c^	Pilot plant	1000 t per year	^182^ and ^183^

a P-Terephthalic acid.

b No information available.

c Bis(2-hydroxyethyl) terephthalate.

d Dimethyl terephthalate.

e 1,8-Diazabicyclo[5.4.0]undec-7-ene.

f Activated carbon.

g Suitable for applications in composites, adhesives, foams, coatings, plasticizers industries.

Polyamide-6 recycling into caprolactam has been commercialized by Aquafill. Although the exact process is unknown, it is referred to as a thermal process, processing pre- and post-consumer products such as fishnets, carpets, and waste generated by polymer industries.^173^

## Chemical and enzymatic recycling of emerging biopolymers

Poly(lactic acid) (PLA) is nowadays one of the most successful bio-based plastics with an estimated production volume of around 190 kilotons in 2019.^184^ Following starch blends, it is the bio-degradable polymer used in highest volumes.^185^ Besides biodegradation by microbes at industrial composting conditions, PLA can also be degraded by hydrolysis or alcoholysis, photodegradation, and enzymatic degradation.^185–187^ The complete hydrolysis of PLA is considered economically not feasible due to high energy costs to remove water and the racemization of l-lactic acid into d-lactic acid. Also alcoholysis of PLA is not stereospecific.^187^ Despite these drawbacks, Natureworks has developed a hydrolysis process that has been hydrolysing 17 million pounds of off-grade PLA resin since 2004.^188^

Another partially biobased polymer which has gained interest in the last decade is poly(butylene succinate) (PBS), which is currently produced from biobased succinic acid and fossil-based 1,4-butanediol. The chemical recycling of PBS has not been studied in great detail. For example, hydrolysis of PBS using 1 M NaOH at 25 °C only resulted in a maximum weight loss of around 8 wt%.^189^ In contrast, enzymatic degradation of PBS is well-studied. Initial efforts focussed on depolymerisation using lipases, which typically takes multiple days to reach completion.^190,191^ A recent report by Bai *et al.* described the depolymerisation of PBS films with a cutinase similar to those applied for PET depolymerisation, achieving 90% weight loss of a PBS film sample in 24 h.^192^

The polyester poly(ethylene furanoate) (PEF) is widely advocated as a renewable alternative to PET in many application areas. The first report on the synthesis of PEF dates back to 1946 by British Celanese,^193^ but interesting properties like permeability and other physical/chemical characteristics were only explored in recent decades.^194^ Up till now, several hundred scientific articles and patents have been published on 2,5-FDCA based polyesters derived using different co-monomers. The majority of the scientific reports address the improvement of the properties of furanic polyesters to a level comparable to those of its aromatic analogues such as PET and PBT, with the aim of replacing terephthalic acid by 2,5-FDCA either partially or fully depending on the performance, cost and availability of this renewable monomer in the future.^195–197^ Although the market volume of PEF is currently very small (or not yet) compared to PET, it is important to investigate the depolymerisation potential of PEF, preferably by breaking it down to its monomers 2,5-furandicarboxylic acid and EG, as a the recycling method.

The depolymerisation of furanic polyesters has been investigated by employing the conditions that are known to work for PET depolymerisation. These include hydrolysis, methanolysis and glycolysis processes in the presence of a catalyst.^198^ For example, poly(butylene 2,5-furandicarboxylate) (PBF) with a particle size of 0.6–1.4 mm, and *M*_n_ 17 kg mol^−1^ was depolymerized using 25 wt% NaOMe solution at 90 °C for 2.0 h, yielding 60.6% of the monomer 2,5-furandicarboxylate. Similarly, PEF with *M*_n_ 15 kg mol^−1^ and a particle size of 0.6–1.4 mm, was hydrolyzed using 2.5 M NaOH solution at 100 °C for 6.0 h. After neutralization with HCl, 82.9% yield of 2,5-furandicarboxylic was obtained. The depolymerization was also investigated using 1,5,7-triazabicyclo[4.4.0]dec-5-ene (TBD) a bicyclic strong, soluble organic base. This yielded 63.1% of the monomer which is lower than obtained with sodium methoxide. The methanolysis rate of PEF and PET tensile bars was investigated individually at 90 °C under similar conditions. It was recorded that faster depolymerisation of PEF was observed compared to PET, with around 52 and 2% dissolution after 90 minutes, respectively.^198^

Ribitsch *et al.* investigated the enzymatic hydrolysis of FDCA-derived polyesters based on the carbon chain length of the incorporated diols (*n* = 2, 3, 5, 6, 8, 9, or 12). For this hydrolysis, the enzyme cutinase 1 from *Thermobifida cellulosilytica* (Thc_Cut1) was employed at 50 °C for 72 h at pH 7.0. These conditions were previously shown to be suitable for PET hydrolysis as well.^84,199^ Thc_Cut1 hydrolyzed all the tested polyesters, displaying the highest activity towards 1,5-pentanediol and 1,9-nonanediol-based polyesters, whereup to 58% to FDCA released. Moreover, the authors also observed increased activity when replacing the linear 1,3-propane diol with 1,2-propane diol or by introducing ethoxy units in the polyester chain.^200^ In parallel, the influence of ionic moieties and polyol structures on the hydrolytic stability of 2,5-FDCA based co-polyesters was investigated. In addition to the previously observed trend related to varying the chain length of the diol, the presence of an ionic phthalic acid in the backbone also had a positive effect. The enzyme Thc_Cut1 is capable of hydrolysing polyesters with a preferred cleavage of ester bonds in the vicinity of the non-charged building blocks.^201^

The effect of molecular mass, particle size and crystallinity of the PEF polyester (powders and amorphous films) on enzymatic hydrolysis was investigated using different cutinases from *Thermobifida cellulosilytica* (Thc_Cut1) and *Humicola insolens* (HiC). The enzyme Thc_Cut1 efficiently hydrolysed PEF powders with low molecular masses (*M*_n_ 18 kg mol^−1^) and low crystallinity compared to higher MWs (*M*_n_ 55 kg mol^−1^) and higher crystallinity.^202,203^ It was also observed for two different molecular weights that the particle size (180 < *d* and 180 < *d* < 425 μm) does not have any significant impact on depolymerization.

Both enzymes, Thc_cut1 and HiC were investigated for the degradation of amorphous PEF films at different temperatures (50 and 65 °C) and different conditions, *i.e.*, 0.1 M Tris–HCl at pH 7 and 1 M KPO pH 8. Of the two enzymes, HiC showed superior hydrolytic activity compared to Thc_cut1 with 100% degradation of PEF film initially to soluble molecules (oligomers) and further degradation to monomers over time, as identified by LC-MS/TOF analyses.^202^

## Discussion and future perspectives

A future circular economy mandates an increased use of renewable polycondensation polymers at the expense of fossil-based polymers or even renewable based polyolefins. Renewable polycondensation polymers can be produced more efficiently from biomass (or CO_2_) than renewable based polyolefins, in terms of atom economy, required conversion processes and overall life cycle assessment. Furthermore polycondensation polymers can be more effectively be (chemically) recycled than polyolefins: the current yields of back to original monomer (this implies that the polymer is recycled into the same monomer(s) as the original building block(s)) chemical recycling of the major polycondensation polymer, PET is already in the order of >90%. Back to original monomer chemical recycling is also very effective for polystyrene; recovery of the monomer styrene can be over 70%.^204–206^ Chemical recycling of polyolefins, for example polyethylene or polypropylene, on the other hand, performed *via* processes like pyrolysis under an inert atmosphere at temperatures ranging typically from 400–900 °C, yields a mixture of oils, gases and a char or wax fraction.^207,208^ The mixture of compositions obtained depend on the feedstock and operating conditions. Yields of valuable monomers (*e.g.* ethylene, propylene, butenes, BTX) suitable for polymerisation can be substantial (even over 70%), but the yields original monomers are moderate. This complicates the whole recycling operation, since the individual products must be separated from complex mixtures and multiple product outlets need to be pursued.

Given the nature of the carbon–carbon bond in polyolefins and the high temperatures required for their chemical recycling, it is unlikely that a similar yield of back to original monomer recycling can be achieved as for polycondensation polymers. Thus, production of new polymer building blocks by chemical recycling requires a much higher input of virgin feedstock for polyolefins than for polycondensation polymers, making polycondensation polymers superior from a circularity point of view.

Whereas due to current production volumes and existing assets, high production of recycled polyolefins might be anticipated up to 2050–2060, a gradual replacement of polymers by polycondensates should occur in a circular economy, with polycondensates replacing polyolefins as the major polymer class by 2050–2060.

Such future circular polycondensation polymers may comprise both drop-in products, like recycled or biobased PET and new polymers like PEF, PLA, PBS, PHA's and other biobased polycondensation polymers; in addition to polyesters they will likely also comprise renewable-based polyamides and/or polycarbonates.

Recycling of these polycondensation polymers will occur preferably by mechanical recycling, or, in cases where this is not possible, *via* chemical or biochemical recycling. In the coming years, chemical recycling of polycondensation polymers will be further explored and optimised in terms of yield of monomer recovered, process conditions, catalyst loading and downstream processing and purification of the resulting monomers. Mild inorganic catalysts might even introduce the possibility of selective depolymerisation of one polycondensation polymer in the presence of another, which is a benefit in multi-polymer fractions. Biochemical recycling using (isolated) enzymes is currently not yet as efficient as chemical depolymerisation, but huge opportunities exist for the improvement of current enzymatic systems. Due to their mild window of operation, even milder than for inorganic catalysts, they offer the opportunity to hydrolyse one polymer in the presence of another, enabling selective recovery of monomeric building blocks from mixed polymer fractions. These mild reaction conditions are also of interest from the point of the low associated energy use, particularly considering the likelihood that (further) legislation aimed at reducing the carbon footprint of industrial processes will be introduced in future.

Furthermore, enzyme-catalysed depolymerisation offers the opportunity to include packages of degrading enzymes in the polymer, thereby inducing (bio)degradability at the appropriate moment. In addition, knowledge about polymer-degrading enzymes, will teach us which polymers can be degraded in the environment and will help us to design polymers that are “circular by design”; renewable based polymers that can be efficiently recycled and in addition have the ability to biodegrade if they unintentionally end-up in the environment.

## Conflicts of interest

There are no conflicts to declare.

## Supplementary Material
